# Dopamine release in mushroom bodies of the honey bee (*Apis mellifera* L.) in response to aversive stimulation

**DOI:** 10.1038/s41598-018-34460-1

**Published:** 2018-11-02

**Authors:** David Jarriault, Justine Fuller, Brian I. Hyland, Alison R. Mercer

**Affiliations:** 1 Department of Zoology, University of Otago, Dunedin, New Zealand; 2 Department of Physiology, Otago School of Biomedical Sciences, University of Otago, Dunedin, New Zealand

## Abstract

In *Drosophila melanogaster*, aversive (electric shock) stimuli have been shown to activate subpopulations of dopaminergic neurons with terminals in the mushroom bodies (MBs) of the brain. While there is compelling evidence that dopamine (DA)-induced synaptic plasticity underpins the formation of aversive memories in insects, the mechanisms involved have yet to be fully resolved. Here we take advantage of the accessibility of MBs in the brain of the honey bee to examine, using fast scan cyclic voltammetry, the kinetics of DA release and reuptake *in vivo* in response to electric shock, and to investigate factors that modulate the release of this amine. DA increased transiently in the MBs in response to electric shock stimuli. The magnitude of release varied depending on stimulus duration and intensity, and a strong correlation was identified between DA release and the intensity of behavioural responses to shock. With repeated stimulation, peak DA levels increased. However, the amount of DA released on the first stimulation pulse typically exceeded that evoked by subsequent pulses. No signal was detected in response to odour alone. Interestingly, however, if odour presentation was paired with electric shock, DA release was enhanced. These results set the stage for analysing the mechanisms that modulate DA release in the MBs of the bee.

## Introduction

The biogenic amine dopamine (DA) is a ubiquitous neuromodulator across many animal species, involved in functions as diverse as locomotion^1,2^, arousal^3,4^, learning and memory^5–8^ and decision-making^9,10^. DA neurons in the insect brain are found in discrete clusters that project into specific brain regions, including into areas known to play a critical role in learning and memory formation^11,12^. In the fruit fly, *Drosophila melanogaster*, subpopulations of DA neurons projecting to the mushroom bodies (MBs) of the brain have been identified that respond to noxious (electric shock) stimuli^11,13,14^. Using a classical conditioning paradigm, activation of these neurons can substitute for the unconditioned aversive stimulus^8,13,15^, whereas blocking output from these neurons^16^ or inhibiting DA receptor function^17–19^, has been found to impair aversive memory. Despite rapid advances in this area^20,21^, the kinetics of DA release and reuptake, and mechanisms that underlie the modulatory actions of DA in MBs of the brain have yet to be fully resolved.

**Figure 1:**
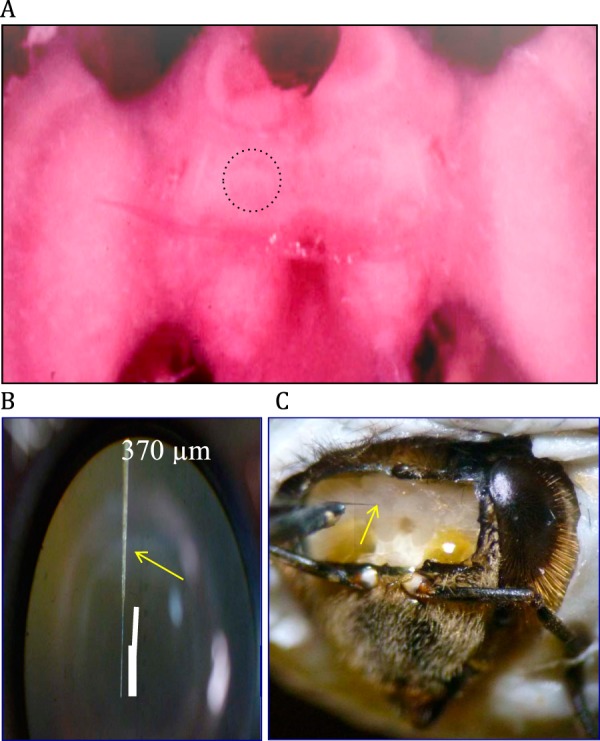
Brain of the honey bee, *Apis mellifera*. (**A**) Brain stained with neutral red to show the position of the tips of the vertical lobes of the mushroom bodies. The boundary of the distal tip of the right vertical lobe is indicated. (**B**) Recording electrode. (**C**) Placement of the recording electrode (arrow) in the right vertical lobe.

Here, we take advantage of the size and accessibility of MBs in the brain of the honey bee (*Apis mellifera* L.) to record electrochemical responses in the MBs elicited by electric shock. In honey bees, the vertical lobes of the MBs project anteriorly with regard to the body axis^22^. A window cut in the head capsule above a bee’s antennae reveals the tips of the vertical lobes, which are visible on the surface of the brain (Fig. 1). In this study, recording electrodes are inserted parallel to the projections of MB intrinsic neurons (Kenyon cells) that form the vertical lobe, and fast scan cyclic voltammetry (FSCV) is used to monitor changes in the extracellular concentration of electroactive molecules in this well-defined MB neuropil. FSCV enables biogenic amines, such as DA, to be identified specifically, and detected *in vivo* with high sensitivity and sub-second temporal resolution^23^. This technique has been used extensively in mammals^24,25^ and has recently been applied with success also to invertebrates^26–28^.

Using FSCV we show that DA is released in the MBs in response to noxious stimuli applied to the abdomen of the bee. We describe the kinetics of DA release and reuptake in this well-defined region of the brain, and identify factors affecting the release of this amine in response to electric shock. Because insects learn rapidly to associate odour with punishment^29,30^ and this association has been shown in *Drosophila* to require the coincident activation of MB Kenyon cells and DA neurons projecting to the MB neuropil^31,32^, we examine also the effects of odour alone, and odour delivery coincident with electric shock. Our results reveal that odour paired with electric shock modulates DA release in MBs of the brain.

## Results

### Dopamine release in response to electric shock

**Figure 2:**
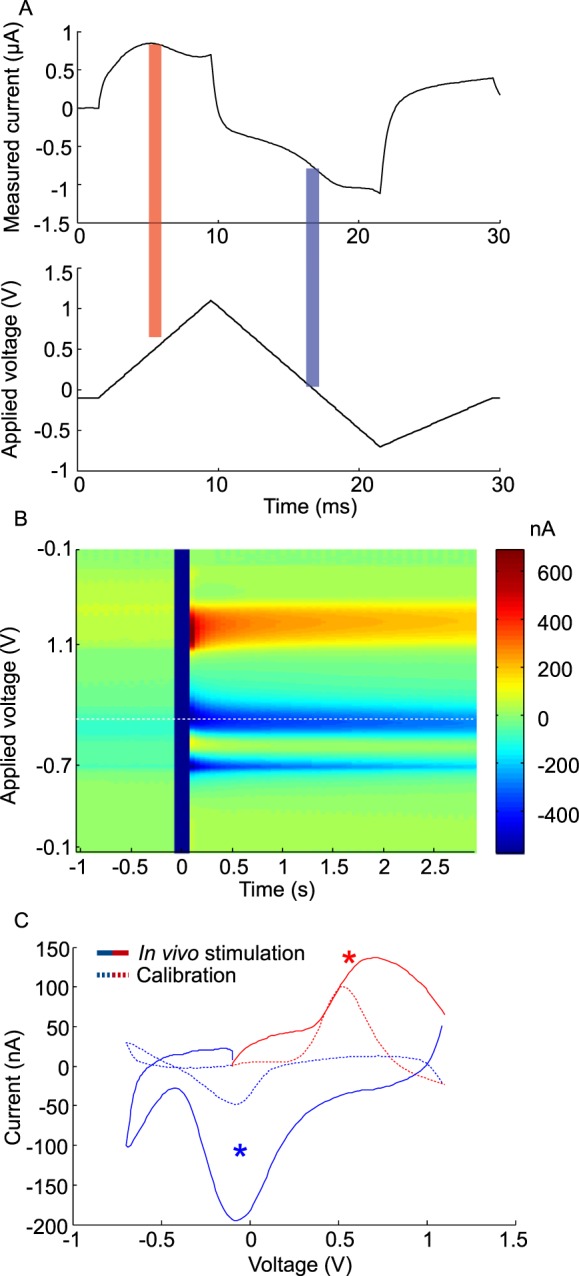
Signals in the vertical lobe of the MBs evoked by electric shocks. (**A**) Voltage ramp applied to the recording electrode (lower trace) and resulting current measured (upper trace). Oxidation and reduction peaks are indicated by a red and a light-blue bar, respectively (**B**) Colour plots of the voltammogram. Vertical dark-blue bar around time point zero represents the electrical stimulation (100 ms, 10 V) delivered to the abdomen of the bee. The dashed white line denotes the reduction potential (−0.1 V) utilised for the signal traces shown in Fig. 3A. (**C**) Cyclic voltammogram measured in response to the shock (solid line) matches the trace obtained during calibration with a 2 µM-DA solution (dashed line). Red and blue lines in Fig. 2C represent the oxidation and reduction portions of the applied voltage sweep, respectively. The red and blue asterisks identify the oxidation and reduction peaks, which correspond to the red and blue areas in the colour plot in (**B**) and the red and blue bars in Fig. 2A.

**Figure 3:**
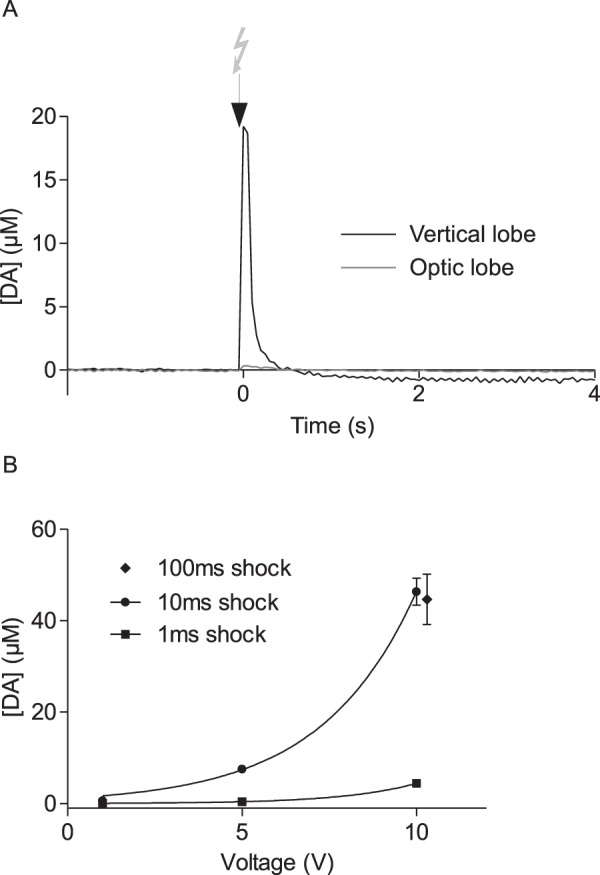
Characterisation of DA signals evoked by stimulation with electric shock. (**A**) Representative signal trace showing the change in DA concentration in the vertical lobe of the MBs over time in response to a 10 V, 100 ms electric shock applied to the abdomen of the bee. Current was converted to DA concentration based on comparisons with electrode calibration data. Black arrow indicates the time at which the electric shock (10 V, 100 ms) was delivered. No dopamine release was detected in the lobula of the optic lobe in the same bee. (**B**) DA release measured in the vertical lobe in response to electric shock stimuli 1 ms (filled squares),10 ms (filled circles) or 100 ms (filled diamonds) in duration, at different intensities (1, 5 or 10 V). Data points (mean DA concentration ± SEM) were fitted with exponential curves. Data presented in this graph represent 314 measurements from 33 bees.

Voltage ramps were applied to the recording electrode (Fig. 2A, lower trace) and the resulting current measured (Fig. 2A, upper trace). Background-subtracted cyclic voltammograms were derived (e.g. Figure 2C) and data were visualised as false colour plots showing delta current at all scan voltages over time (e.g. Figure 2B), or as concentration *vs*. time traces at a particular scan voltage representing DA concentration (e.g. Figure 3A). Identification of the amine released was based on oxidation and reduction peaks measured in calibration voltammograms (e.g. Figure 2C, dashed line). As the oxidation peak of the biogenic amine serotonin exhibited overlap with that of DA (supplementary Fig. S1), DA concentration was quantified from the change in current observed during the reduction peak, which exhibited a unique (DA-specific) signature. Changes in amplitude of the reduction peak were reflected proportionally by changes in the size of the oxidation peak.

In response to a brief electric shock (100 ms, 10 V), background-subtracted cyclic voltammograms showed a strong change in current in the MBs which, when compared with the calibration data (Fig. 2; supplementary Fig. S1), displayed the characteristic signature of DA. Current induced by stimulation with electric shock increased close to 0.6 V and decreased close to −0.2 V, respectively (Fig. 2B,C). Similar signals in response to electric shock were apparent also in the antennal lobes (data not shown), but could not be detected in the lobula of the optic lobe (Fig. 3A). No variation in current was detected at the reduction potential for serotonin (0.2 V, suppl. Fig. S1) or at the oxidation potential recorded for octopamine (0.9 V, suppl. Fig. S1), suggesting that neither serotonin nor octopamine is released in the vertical lobe of the MBs in response to stimulation with electric shock.

DA concentration was calculated from the current variation measured in response to DA standards tested during electrode calibration. The concentration *vs*. time trace in Fig. 3A shows changes in DA were time-locked to the stimulus. Using 10 V, 100 ms stimuli, maximum release occurred on average 96 ± 12.8 ms (mean ± SEM; n = 20) after the electric shock onset and ranged from 9 to 102 µM, with an average value of 44.6 ± 5.5 µM (mean DA release ± SEM). The signal returned to 25% of the maximum after 472.2 ± 135.6 ms (mean ± SEM). To estimate the kinetics of clearance, the decay portion of each peak was fitted with a single exponential decay function of the following form:

$$[{\rm{DA}}]({\rm{t}})={[{\rm{DA}}]}_{{\rm{\max }}}\ast {e}^{-{\rm{k}}\ast {\rm{t}}}$$

where [DA] is the DA concentration at a given time, [DA]_max_ is peak concentration, and k is the first-order rate constant^33^. The exponential constant k, representing the DA clearance rate ranged from 0.02 to 0.93, with an average of 0.44 ± 0.06.

### Stimulus parameters influence DA release

Three voltage intensities were tested to examine the relationship between shock intensity and DA release. DA release was strongly dependent on the voltage of the shock applied (Fig. 3B). Using 10 ms stimuli ranging from 1 to 10 V, the concentration of DA measured increased from 0.6 ± 0.1 µM in response to a 1 V stimulus up to 46.3 ± 2.9 µM in response to a 10 V stimulus. DA release was also dependent on the duration of the electric shock. Using 10 V stimuli, we found approximately 10 times more DA was released in response to a 10 ms stimulus (46.3 ± 2.9 µM) than in response to a stimulus 1 ms in duration (4.5 ± 0.9 µM; suppl. Fig. S2). Stimuli lasting 100 ms, however, resulted in DA signals that were similar to those measured in response to 10 ms stimuli (mean DA release ± SEM: 44.6 ± 5.5 µM versus 46.3 ± 2.9 µM, respectively). As electrodes were capable of responding incrementally to increasing DA concentrations up to at least 160 µM, our results suggest that 10 V, 10 ms stimuli elicit maximum activation of dopaminergic neurons projecting to the MBs of the honey bee.

### DA release and behavioural responses to shock

**Figure 4:**
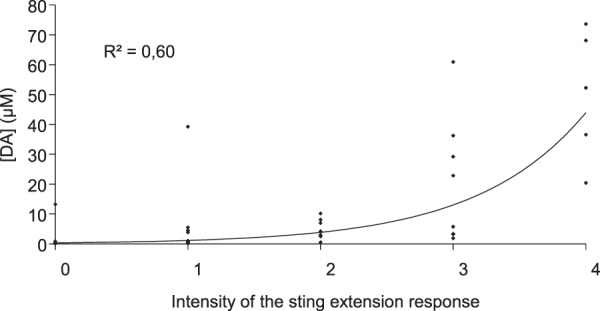
Relationship between DA signals and behavioural responses to electric shocks. Shock stimuli ranging in intensity from 1, 5 and 10 V, and 1 and 10 ms in duration are included in the analysis. Behavioural responses were classified as follows: 0 = no movement or sting extension, 1 = weak abdominal movement but no sting extension, 2 = strong abdominal movements but no sting extension, 3 = weak sting extension, 4 = strong sting extension. Data points were fitted with an exponential curve. Data presented in this graph represent 42 measurements from 17 bees.

In bees, electric shock elicits a defensive sting extension reflex. We therefore examined whether there was a quantitative relationship between the magnitude of the sting extension response and the quantity of DA released in the MBs. Behavioural responses were classified, in order of magnitude, into 5 categories: (1) *no movement or sting extension*; (2) *weak abdominal movements without sting extension*; (3) *strong abdominal movements without sting extension*; (4) *weak sting extension*; (5) *strong sting extension*. At the population level, DA release and behavioural response amplitude exhibited an exponential relationship (correlation coefficient of the exponential fit to the data: R^2^ = 0.6). However, exceptions were noted. In some cases, the sting was extended at times when very low levels of DA release were recorded and in other instances, DA release occurred in the absence of any extension of the sting (Fig. 4).

### Pharmacological manipulation of DA clearance

**Figure 5:**
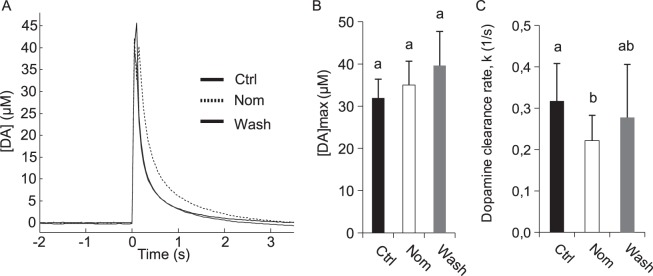
Pharmacological manipulation of DA reuptake. Nomifensine (800 µM), a DA reuptake inhibitor, was added to the brain perfusate 10 min prior to stimulation with electric shock (10 V, 100 ms). (**A**) Representative traces recorded before treatment (Control, Ctrl), after 10 min exposure to nomifensine (Nom) and following a 10 min saline wash (Wash). (**B**) Maximum DA release (mean + SEM). Nomifensine did not alter the amplitude of DA release. (**C**) Clearance rate (mean + SEM). Nomifensine (800 µM) slows DA reuptake. Data presented in this figure represent 55 measurements from 5 bees.

The electrochemical experiments described above strongly suggest that the observed signal arises from DA release. To confirm this, we manipulated DA signaling in the honey bee by treating animals with nomifensine, a potent inhibitor of the insect DA transporter (DAT^34^). When added to the saline solution bathing the brain, nomifensine (800 µM) increased the time required for the DA signal to disappear. In Fig. 5A, representative traces obtained from a single individual before and after treatment with 800 µM nomifensine, together with the after-wash signal, are superimposed to highlight this delay. The peak concentration was not significantly affected by the drug (Friedman’s test: Q = 1.2, p = 0.55, Fig. 5B). However, after nomifensine treatment, DA clearance rate, as represented by the rate constant, k, was decreased 1.45-fold from 0.32 ± 0.09 to 0.22 ± 0.06 (Friedman’s test: Q = 6.4, p = 0.039, *post-hoc* comparison using Dunn’s test; Fig. 5C).

### HVA and DA release

**Figure 6:**
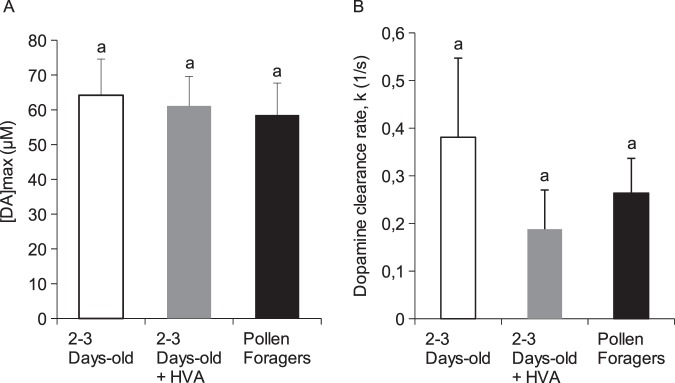
Comparison of DA release evoked by shock stimuli (10 V, 100 ms) recorded in 2 to 3-day old workers versus pollen foragers. (**A**) Maximum DA release (mean + SEM). (**B**) Clearance rate (mean + SEM). Treatment of young bees with 100 µM HVA had no significant effect on DA release in MBs of the brain. DA release in young bees was similar to that observed in pollen foragers. Data presented in this figure represent 109 measurements from 3 groups of 10 bees.

Earlier studies have shown that aversive learning in young worker honey bees can be suppressed by exposing bees from the time of their emergence as adults to queen mandibular pheromone, or to one of its key components, homovanillyl alcohol (HVA)^35^. Because effects of HVA on DA release in the MBs could potentially explain the inhibitory effects of this pheromone on aversive learning, we compared shock-induced DA release in young (2- to 3-days old) control (untreated) bees with DA release in bees of the same age treated with HVA. DA release in pollen foragers, which are generally around 3 weeks of age or older, was also examined. No significant differences, either in the maximum levels of DA release (ANOVA: F_(2,27)_ = 0.09; p = 0.91; Fig. 6A) or in the clearance rate constant, k (Kruskal-Wallis: K = 1.44, p = 0.49; Fig. 6B), were identified between the 3 groups.

### Response amplitude and shock repetition

**Figure 7:**
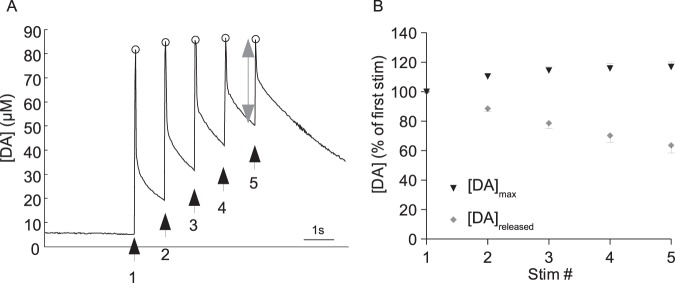
Signals evoked by 5 successive electric shock stimuli (100 ms, 10 V) presented at 1 s inter-stimulus intervals. (**A**) Trace shows changes in DA concentration over time. Circles signal the maximum DA concentration recorded in response to each stimulation. The arrows show the timing of successive stimulations. The double-headed arrow shows the concentration of DA released in response to the fifth stimulation. (**B**) Responses normalised to the DA signal recorded in response to the first stimulation, and averaged across individuals. The maximum concentration of DA recorded in response to each stimulus ([DA]_max_) is shown, along with the concentration of DA released in response to each successive stimulation ([DA] released). Data presented in this figure represent 89 measurements from 23 bees.

To investigate DA release in response to repeated stimuli, bees received 5 electric shock stimuli presented 1 s apart. In all of the animals tested for this experiment (n = 23), each of the 5 stimuli evoked a discrete DA signal (*e.g*. Figure 7A). While peak DA levels increased as a result of repeated stimulation (ANOVA for repeated measures: F_(4,88)_ = 17.25; p < 0.001), post-hoc analyses revealed a significant increase only between responses to the first two stimuli. At this frequency, DA levels were unable to return to baseline between each stimulus, and with each stimulus the amount of additional DA released (measured as the difference from the immediate pre-stimulus level to the peak) decreased (ANOVA for repeated measures: F_(4,88)_ = 39.97; p < 0.001; Fig. 7B).

### Odour presentation coincident with electric shock

**Figure 8:**
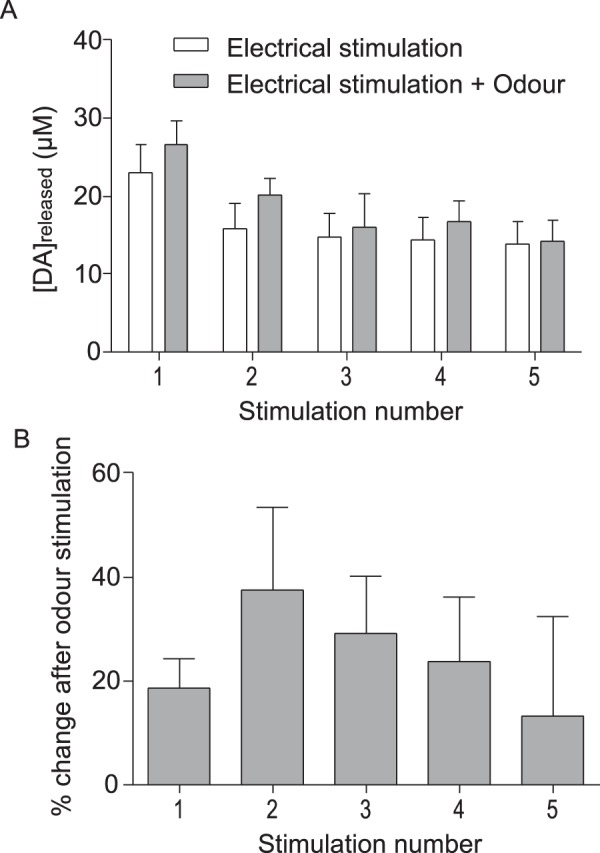
Effects of presenting a novel sensory stimulus (odour) coincident with electric shock. Trains of 5 electric shock stimuli (100 ms, 10 V) were presented 1 s apart followed by a 1 minute recovery period. (**A**) Dopamine concentration released in response to electric shock stimuli presented without odour stimulation (white bars) compared to the responses observed when bees were presented with shock accompanied by odour stimulation of the antennae (grey bars). (**B**) Percent difference between DA released in response to 5 presentations of electric shock alone and in response to the same number of electric shock stimuli presented together with odour stimulation of the antennae. Each set of stimuli (first without and then with odour) was presented twice to each bee. The percent change in response to electric shock resulting from coincident presentation of odour was calculated with each bee serving as its own control. Data presented in this figure represent 38 measurements from 5 bees.

Bees rapidly learn to associate odours with electric shock. To determine whether presentation of a salient sensory stimulus, such as odour, coincident with electric shock affects the magnitude of DA released, odour stimuli were paired with electric shock. In these experiments, 5-shock electrical stimulations were repeated at least 3 times on each bee under control conditions (without odour) and with a 90 second inter-trial interval. The responses to each 5-shock stimulation were similar to those shown in Fig. 7. With each repetition of the 5-shock stimulus, responses remained stable (see Suppl. Fig. S3). In the absence of odour, there was no evidence of response decline, or of response enhancement. The same bees were then presented with the same form of stimulation (5-shock stimuli) repeated at least 3 times with a 90 second ISI, however, this time stimulation with electric shock was accompanied by odour stimulation of the antennae. Figure 8A shows the averaged responses of repetitions with shock alone (white bars) and the averaged responses of repetitions with shock presented together with odour (grey bars). Both signal amplitude (Fig. 8A) and the percent change in signal amplitude resulting from coincident odour presentation (Fig. 8B) were calculated for each bee. We found odour presented together with electric shock significantly enhanced DA release (Fig. 8A, ANOVA for repeated measures: F_(1,20)_ = 13.81; p < 0.05). However, as described above (Fig. 7B), with 5-shock stimulation, the amount of additional DA released with each successive stimulus declined (Fig. 8A). No signal was detected in the MBs in response to odour alone.

## Discussion

Signals detected in the MBs of the bee in response to stimulation of the abdomen with electric shock displayed a signature characteristic of the biogenic amine, DA, indicating that in bees, as in flies^13^, noxious stimuli activate DA neurons projecting to the MBs of the brain. Effects of the DA transport inhibitor, nomifensine, are consistent with this conclusion as nomifensine, which slows DA reuptake^34^, was found to increase the time required for signals in the MBs elicited by electric shock to disappear. This provides the first direct measurement of DA release in MBs of the insect brain. This study is the first also to reveal the kinetics of DA release and reuptake in this well-defined brain region and to show that salient sensory signals can modulate DA release.

Interesting parallels can be drawn between the results of the present investigation and measurements of DA release in the ventral nerve cord and protocerebrum of the fly^27,33,36^. In bees, as in flies, the amount of DA released varied between individuals and was strongly dependent on the stimulation parameters used to evoke release. However, concentrations of DA released in the MBs of the honey bee in response to stimulation with electric shock are 10 to 20 times higher than the DA transients that have been detected in the central nervous system of the fruit fly. It is as yet unclear whether this reflects differences in the methodological approaches applied, the specific region recorded from, or species-specific differences. In the present study, DA release in bees was elicited by sensory stimulation, whereas in prior work in *Drosophila*, DA transients have been evoked by photoactivation of DA neurons expressing either the transgene ChannelRhodopsin 2^27,33,37^, or the cation channel, CsChrimson^36^. The consequences of using different methodological approaches to evoke DA release are currently unknown. Further, while recording electrodes of 250 µm or more in length were used in the present investigation, much shorter electrodes (e.g. 40 to 60 µm) have been used in *Drosophila*^27^. The impact of differences in electrode length could be significant; length determines the effective surface area and the quantity of chemicals that can interact with the carbon fibre and can therefore affect electrode sensitivity.

Regional or interspecies differences in the density of DA terminals in the nervous system of bees and flies could also account for differences in the concentration of DA signals detected. While there are no data as yet relating to interspecies differences, regional differences in DA kinetics have been identified both in the mammalian brain^38^ and recently also in the nervous system of the fly^36^. Using optogenetic stimulation of DA neurons in larval *Drosophila*, Privman and Venton found the amount of DA released per stimulation pulse was higher in the ventral nerve cord of the fly than in the dorsal medial protocerebrum^36^. Maximum clearance rate, on the other hand, was found to be higher in the protocerebrum than in the ventral nerve cord^36^.

Riemensperger and collaborators were the first to demonstrate that DA neurons with terminals in the MBs respond to electric shock^13^. They used calcium imaging, a technique used extensively in the fly to explore the functional properties of DA neurons and their contribution to associative olfactory learning and memory. Calcium signals in DA neurons with terminals in the MBs of the fly are reported to increase linearly with electric shock intensity^11^. In the present study, however, increasing the intensity of electric shocks delivered to the abdomen of the bee resulted in an exponential increase in DA release, a response more similar to the nonlinear relationship between stimulus intensity and DA release from rat midbrain dopaminergic neurons^39,40^.

In the current investigation, 10 V shocks were generally delivered for a duration of 100 ms. Stimuli of this magnitude and duration were chosen because they reliably elicit sting extension. However, our data show that DA neurons with terminals in the MBs are activated maximally if 10 V stimuli are applied for any duration longer than 10 ms. Stimuli of a similar intensity applied for a much longer duration (generally 2 s), have been used as negative reinforcement in studies of aversive learning in honey bees^30,35,41^. Although the behavioural responses to electric shock appear similar, stimuli of as short a duration possible would seem preferable if electric shock is to be used in the future as the unconditioned stimulus in an aversive conditioning paradigm.

Our results show that with repeated stimulation, the amount of DA released in response to the first stimulus is generally higher than amounts detected in response to subsequent stimuli. Response heterogeneity similar to that observed in the present investigation has been described recently both in the mammalian brain^42^, as well as in the central nervous system of the fly^36^. One explanation is that DA release is controlled by autoregulation. In vertebrates, D2 DA receptors located either on the axon terminals, or on the somatodendritic area of DA neurons^43,44^, serve to regulate the synthesis and release of DA^45,46^. D2-like DA receptors have been identified also in insects^47,48^ and involvement of the D2-like receptor, DD2R, in controlling DA release has recently been demonstrated in the fly^37^.

Expressed *in vitro*, the honey bee orthologue of DD2R (AmDOP3) can be activated by a component of queen mandibular pheromone, HVA^49^, an aromatic compound found to lower brain DA levels and to suppress aversive learning in young worker honey bees^35,50,51^. This, together with evidence implicating DD2R in the control of DA release in the fly^37^, suggested to us the possibility that HVA’s effects on aversive learning in the bee might result from HVA-induced changes in DA release at the level of the MBs of the brain. While this possibility requires further investigation, we found no evidence to support this hypothesis. Moreover, despite evidence that brain DA levels are higher in foragers than in young worker bees performing tasks within the colony^52,53^, and DA transporter expression increases with age in the honey bee brain^54^, we found no indication of age-related differences in either the amplitude or clearance rate of shock-evoked DA transients in the MBs of the bee. However, further work is required on bees of known age and behavioural caste.

Of particular interest, is the effect observed in this study of delivering a novel salient sensory stimulus (odour) coincident with electric shock. While odour alone failed to elicit DA release in the MBs, our results show that DA release evoked by electric shock is enhanced if odour presentation is paired with negative reinforcement. This suggests that Kenyon cell activity can modulate the output of DA neurons with terminals in the MB neuropil. Consistent with this possibility, direct synaptic input from Kenyon cells onto DA neurons has recently been identified in the fly^55,56^. MBs are sensory integration centres, receiving and responding to a diverse array of sensory signals^22,57^. Although involved in signal gain control, response sparsening and signal discrimination^58^, MBs are best known for their role in olfactory learning and memory^20^. It has long been known that if an odour is paired with electric shock, insects learn to associate the odour with punishment and respond subsequently with odour avoidance^29^. Associative olfactory learning is underpinned by DA’s ability to modulate Kenyon cell synaptic activity^20,21^. DA receptor coupling to the cAMP signaling pathway, together with the expression in Kenyon cells of a calcium/calmodulin-sensitive form of adenylyl cyclase enables MB neurons to detect and respond to coincidence between olfactory and somatosensory inputs to the MB during olfactory learning^32,51,59–61^. Recently in *Drosophila*, a positive feedback loop involving reciprocal axoaxonic synapses between Kenyon cells and DA neurons has been found to be critical for learning^31^ and there is evidence that coincident activity of Kenyon cells may gate the release of DA from dopaminergic neurons with terminals in the MBs of the fly^32^. Our results are consistent with these findings and reveal for the first time the impact of coincident activity of Kenyon cells on DA release in MBs of the brain.

The results of the present investigation confirm that DA is released in the MBs in response to stimulation with electric shock. The study also describes the kinetics of DA release and reuptake in this important region of the insect brain. Our study reveals in addition that at the level of the MBs, odour stimuli delivered coincident with negative reinforcement modulate DA release. These findings set the stage for analysing the mechanisms that underlie DA release in the MBs of the bee.

## Methods

### Honey bee preparation

Adult *Apis mellifera* L. workers were collected from three colonies housed in hives located in the Department of Zoology at the University of Otago. The forager bees used in this study were pollen foragers, captured at the hive entrance and identified by the presence of pollen baskets on their hind legs. Young bees were also examined. In this case, newly-emerged adult workers were collected from brood frames and maintained in plastic cages (12 cm × 12 cm × 15 cm, 50–60 bees per cage) for 2-3 days. The caged bees were placed in constant darkness in a humidified incubator held at 34 °C and fed *ad libitum* on a mixture of sucrose, pollen and honey.

Prior to each FSCV experiment, bees were chilled on ice until they stopped moving, and then harnessed in cylindrical metal holders. The head and antennae of the bee were fixed in place using wax. The brain was exposed by cutting a small window in the head cuticle and removing the head glands (Fig. 1). The surface of the brain was kept moist with saline solution (NaCl 137 mmol.L^−1^, KCl 2.7 mmol.L^−1^, Na_2_HPO_4_ 8 mmol.L^−1^, KH_2_PO_4_ 1.8 mmol.L^−1^, sucrose 105.2 mmol.L^−1^, CaCl_2_ 2.0 mmol.L^−1^, pH 6.7, 400 mOsm).

### Stimulation with electric shock

Conductive gel was applied to the abdomen of each bee to ensure good connectivity between two stimulating electrodes. The anode was placed in contact with the metal holder in which the bee was mounted and the cathode was located in the abdomen. Electrodes were connected to a stimulator (Pulsar 6i, FHC) and unless stated otherwise, 10 V stimuli were applied for 100 ms with an inter-stimulus interval (ISI) of either 1 min or 1.5 min. All response signals returned to baseline during this period. In some experiments the intensity (voltage) and duration of electrical shock was varied, but stimuli delivered never exceeded 10 V.

### Preparation of recording electrodes

Carbon-fibre electrodes were assembled by sealing a single carbon fibre into a glass capillary. Borosilicate glass capillaries (inside diameter 1.62 mm, outside diameter 3.0 mm; Harvard Apparatus Ltd, Edenbridge, Kent, UK) were pulled using a Narishige puller (Narishige Scientific Instrument Laboratory, Tokyo, Japan), and cut so that the larger part of the tubing was a maximum of 1 cm in length. A single carbon fibre (7 µm OD; Amoco, NJ, USA) was passed through the glass capillary until it protruded about 250–400 µm beyond the capillary tip. A small drop of epoxylite 6001-M (Epoxylite Corporation, Irvine,CA, USA) was then placed inside the glass capillary to seal the tip of the electrode and secure the carbon fibre in place. After baking for 12 hours at 125 °C, the shaft of the glass capillary was filled with Dylon Graphpoxy paste (Dylon Industries Inc., Cleveland, OH, USA), which provided electrical coupling between the carbon fibre electrode and a 5 cm length of enamelled copper wire (0.5 mm OD; Dick Smith Electronics, NZ) embedded also in the graphpoxy paste. The other end of the copper wire was soldered onto a gold-plated amphenol socket (Ginder Scientific, Ottawa, ON, Canada) and connected to an amplifier. Once the glass capillary had been filled with graphpoxy the electrodes were baked for a further 2 hours at 125 °C to harden the paste. Electrode potentials were controlled and changes in current were monitored using a customised pre-amplifier coupled with a CyberAmp 380 amplifier (Axon, USA). Signals were digitised using a Digidata 1400 data acquisition board (Axon, USA) and recorded at a sampling rate of 10 kHz using P-Clamp (Axon, USA).

### Electrode calibration

Electrodes were calibrated prior to each experiment by placing them, together with a standard commercial reference electrode (Quanteon LLC, Nicholasville, KY, USA), in 40 mL of the saline solution used to superfuse the honeybee brain. Four separate 40 µL aliquots of DA (2 mM), serotonin (2 mM) and in some cases, octopamine (2 mM) were prepared in distilled water. After collecting baseline measurements for 30 s, each aliquot of an amine solution was added in turn to the solution used to calibrate the electrode to provide four amine concentrations ranging from 2 to 8 μM. The electrode was washed thoroughly and placed in fresh saline solution between the calibration of different amines. Detection capacities of the electrodes were tested using higher DA concentrations in a calibration series. This revealed that the electrodes were capable of responding incrementally to increasing DA concentrations up to at least 160 μM.

### Voltammetric measurements *in vivo*

For *in vivo* measurements, the recording electrode was inserted into the left or right MB via the vertical lobe (Fig. 1A–C). In each brain hemisphere of the bee, DA-immunoreactive neurons projecting to the vertical lobe of the MB originate from 3 clusters of cell bodies (C1–3)^12^. C1 and C2 lie dorsal and dorsomedial, respectively, to the antennal lobe (i.e. anterior and anterior-medial according to the neuroaxis). Cells in these clusters project to the ventral (neuraxis anterior) layers of the vertical lobe. The ventral and lateral margin of the vertical lobes, and the area that represents the transition between the vertical lobe and the pedunculus, receive input from a large-diametre fibre the cell body of which has yet to be identified. A third cluster of DA-immunreactive neurons (C3), located beneath the lateral calyx, sends projections into posterior (neuraxis dorsal) regions of the vertical lobe neuropil, as well as into the pedunculus and calyces^12^. In this study, the electrode was inserted into the ventral (neuraxis anterior) layers of the vertical lobe. The electrode is likely to have extended as far as the transition between the vertical lobe and pedunculus. Routine histological techniques were used to confirm, *a posteriori*, the position of the recording electrode. Between scans the carbon fibre was held at −0.1 V, but during each scan the electrode was driven to +1.1 V, down to −0.7 V and back to −0.1 V in a triangular fashion at 150 V/s every 50 ms (Fig. 2A, lower trace). The application of this triangular waveform causes oxidation and reduction of chemical species (e.g. DA and serotonin) that are electroactive within this potential range, producing a stereotypical change in current at the electrode (Fig. 2A (upper trace), red bar and blue bar, respectively). After the collection of baseline data for 30 s, bees were subjected to a series of electric shock stimuli, as described above.

### Manipulation of DA release

Treatment with the DA reuptake inhibitor, nomifensine, was used to help confirm the identity of the amine released in the MBs in response to electric shock stimuli. Nomifensine (Sigma-Aldrich) at a concentration of 800 µM was freshly dissolved in saline solution and applied directly to the brain. To allow time for diffusion into the brain tissue, recordings were initiated 10 min after drug application.

Effects on DA release of exposing young bees to the queen pheromone, homovanillyl alcohol (HVA), were also examined. HVA has been shown to reduce brain DA levels and to suppress aversive learning in young worker honey bees^35^. Because HVA-induced modulation of DA release could potentially explain the inhibitory effects of this pheromone on aversive learning (see Discussion), we compared shock-induced DA release in the MBs of young (2- to 3-days old) control (untreated) bees with DA release in bees of the same age treated with HVA. Newly-emerged adult worker bees were placed in cages as described above and provided either with food containing 100 μM HVA (treatment group) or with sucrose, pollen and honey alone (controls). DA release in the young bees was compared also with DA release in pollen foragers, which are typically around 3 weeks of age, or older.

### Responses to shock repetition and shock-odour pairing

To examine the effects of shock repetition, we began by examining bees’ responses to 5 shock stimuli delivered 1 s apart. The same form of stimulation (5-shocks with an ISI of 1 s) was then used to examine the effects of repeated stimulation, first, under control conditions (without odour) and then, in the same bees, with odour paired to electric shock. Under each condition (with or without odour) the 5-shock stimulus was delivered 4 times with an inter-trial interval (ITI) of 90 s between each repetition.

In order to pair odour with electric shock, a puff of odour (2-hexanol) was delivered to the antennae of the bee for 5 s immediately prior to each 5-shock stimulus. The percent change in DA release resulting from odour presentation paired with electric shock was calculated for each individual, thus each bee served as its own control.

### Data analysis

Data were exported from Axograph as text files and processed offline in Matlab using custom-made algorithms. To analyse the effects of repeated stimulation and the effect of coincident odour presentation, ANOVA for repeated measures was used. Nomifensine treatment effects were evaluated using Friedman’s test with Nemenyi’s procedure for *post-hoc* comparisons. Statistical comparisons of DA release in young (2- to 3-day old) bees (control *versus* HVA-treated) and pollen foragers were performed using 1-way ANOVA for comparison of peak release levels, and Kruskal-Wallis tests for analysis of clearance kinetics.

### Animal ethics

All experiments described in this work were undertaken in accordance with the laws of New Zealand regulating scientific research.

## Electronic supplementary material

Supplementary Information

## Data Availability

Datasets generated and analysed during this study are available from the corresponding author upon reasonable request.
