# Supplementary material for: Dopamine release in mushroom bodies of the honey bee (Apis mellifera L.) in response to aversive stimulation
Source: Sci Rep. 2018 Nov 2;8:16277. doi: 10.1038/s41598-018-34460-1 (PMC6214997; doi:10.1038/s41598-018-34460-1)
Supplement: Supplementary file 1 — Supplementary Information [file 41598_2018_34460_MOESM1_ESM.pdf]

Supplementary Material for SREP-18-16817

Dopamine release in mushroom bodies of the honey bee (*Apis mellifera* L.) in response to aversive stimulation

submitted by

David Jarriault, Justine Fuller, Brian I Hyland, Alison R. Mercer

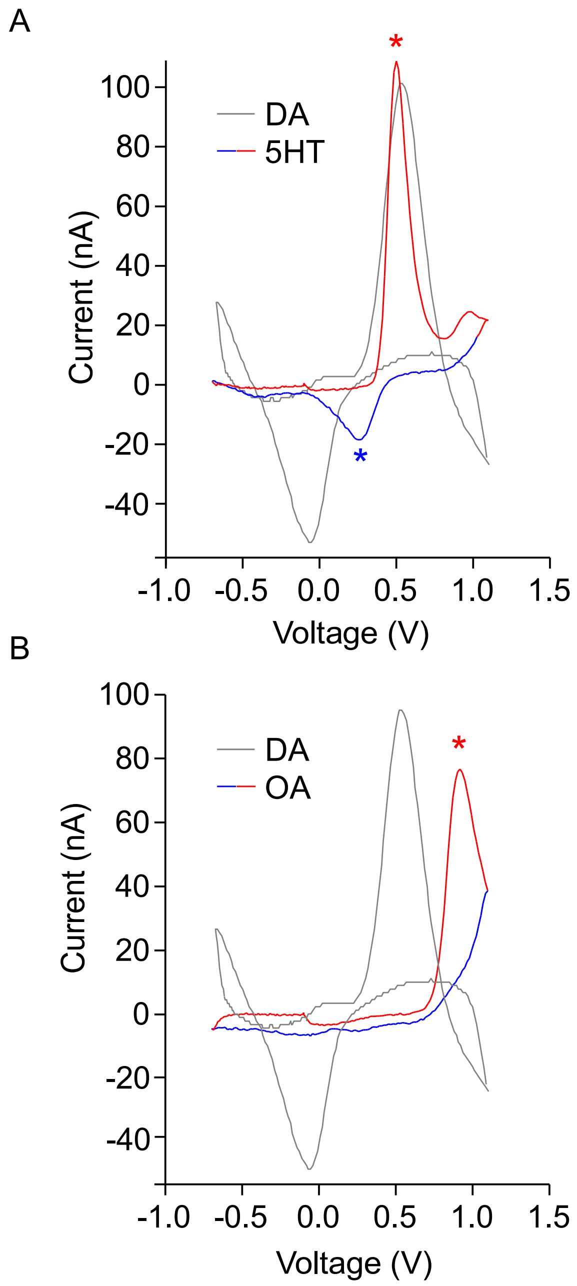

Figure S1: Cyclic voltammograms obtained during electrode calibration with dopamine (DA), serotonin (5HT) and octopamine (OA). (A) Cyclic voltammogram for 5HT (blue and red lines) compared to that of DA (grey line). (B) Cyclic voltammogram for OA (blue and red lines) compared to that of DA (grey line). Red and blue lines represent the oxidation and reduction portions of the applied voltage sweep, respectively. The red and blue asterisks identify the oxidation and reduction peaks. No overlap was observed between DA, 5HT and OA for the reduction current.

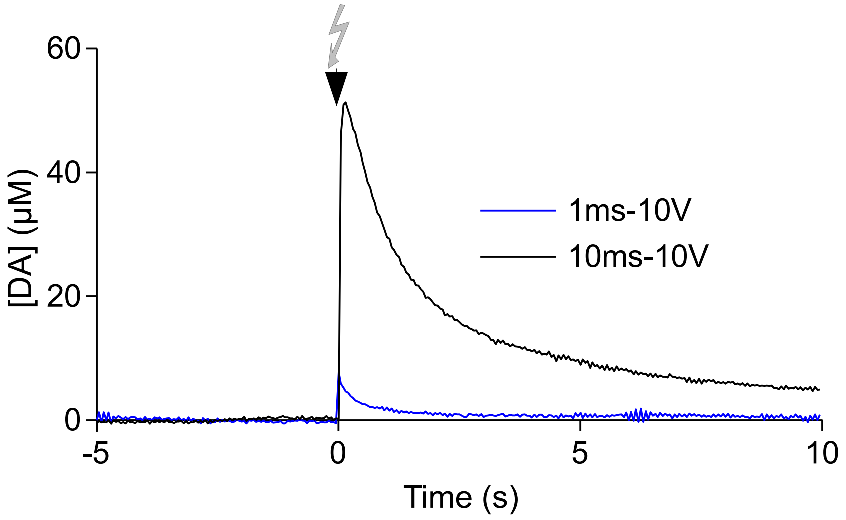

Figure S2 : Representative signal traces showing the change in dopamine (DA) concentration over time in response to two 10 V electric shocks, one applied to the abdomen of a bee for 1ms (blue line) and the second applied for a duration of 10 ms (black line). Current was converted to DA concentration based on comparisons with electrode calibration data.

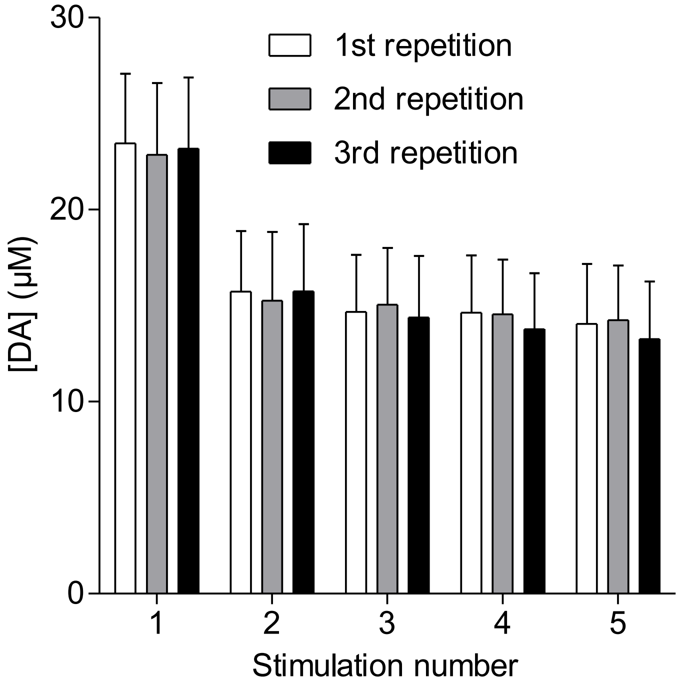

Figure S3: Effect of stimulation repetitions on dopamine release. Three repetitions of the 5-shock stimulus were presented under control conditions (without odour) with a 90 s inter-trial interval. In all repetitions, dopamine release was highest during the first of the five shocks delivered. Overall, however, responses under these conditions were stable, with no evidence of decline or enhancement of dopamine release resulting from repeated stimulation (N = 5 bees).
